# Impact of proton vs. photon radiotherapy on overall survival in the management of spinal chondrosarcoma and mortality risk prediction: A nationwide analysis

**DOI:** 10.1093/noajnl/vdaf240

**Published:** 2025-12-24

**Authors:** Abdul Karim Ghaith, Xinlan Yang, Taha Khalilullah, Anthony Davidson, Yuanxuan Xia, Tej Azad, Jawad M Khalifeh, A Karim Ahmed, Joshua Weinberg, Chase Foster, Nicholas Theodore, Kristin J Redmond, Daniel Lubelski

**Affiliations:** Department of Neurosurgery, School of Medicine, Johns Hopkins University, Baltimore, Maryland (A.K.G., X.Y., T.K., A.D., Y.X., T.A., J.M.K., A.K.A., N.T., D.L.); Department of Neurosurgery, School of Medicine, Johns Hopkins University, Baltimore, Maryland (A.K.G., X.Y., T.K., A.D., Y.X., T.A., J.M.K., A.K.A., N.T., D.L.); Department of Neurosurgery, School of Medicine, Johns Hopkins University, Baltimore, Maryland (A.K.G., X.Y., T.K., A.D., Y.X., T.A., J.M.K., A.K.A., N.T., D.L.); Department of Neurosurgery, School of Medicine, Johns Hopkins University, Baltimore, Maryland (A.K.G., X.Y., T.K., A.D., Y.X., T.A., J.M.K., A.K.A., N.T., D.L.); Department of Neurosurgery, School of Medicine, Johns Hopkins University, Baltimore, Maryland (A.K.G., X.Y., T.K., A.D., Y.X., T.A., J.M.K., A.K.A., N.T., D.L.); Department of Neurosurgery, School of Medicine, Johns Hopkins University, Baltimore, Maryland (A.K.G., X.Y., T.K., A.D., Y.X., T.A., J.M.K., A.K.A., N.T., D.L.); Department of Neurosurgery, School of Medicine, Johns Hopkins University, Baltimore, Maryland (A.K.G., X.Y., T.K., A.D., Y.X., T.A., J.M.K., A.K.A., N.T., D.L.); Department of Neurosurgery, School of Medicine, Johns Hopkins University, Baltimore, Maryland (A.K.G., X.Y., T.K., A.D., Y.X., T.A., J.M.K., A.K.A., N.T., D.L.); Department of Neurosurgery, School of Medicine, Ohio State University, Colombus, Ohio (J.W.); Department of Neurosurgery, School of Medicine, George Washington, Washington, DC (C.F.); Department of Neurosurgery, School of Medicine, Johns Hopkins University, Baltimore, Maryland (A.K.G., X.Y., T.K., A.D., Y.X., T.A., J.M.K., A.K.A., N.T., D.L.); Department of Radiation Oncology, School of Medicine, Johns Hopkins University, Baltimore, Maryland (K.J.R.); Department of Neurosurgery, School of Medicine, Johns Hopkins University, Baltimore, Maryland (A.K.G., X.Y., T.K., A.D., Y.X., T.A., J.M.K., A.K.A., N.T., D.L.)

**Keywords:** machine learning, mortality prediction, photon ­radiotherapy, proton radiotherapy, spinal chondrosarcoma

## Abstract

**Background:**

Spinal chondrosarcomas are rare, aggressive bone tumors with limited data on optimal radiotherapy strategies, particularly regarding the comparison between proton and photon therapies. This study aims to evaluate long-term survival outcomes and identify effective treatments and risk factors using the National Cancer Database.

**Methods:**

Patients diagnosed with spinal chondrosarcomas were categorized into radiation and no-radiation groups. The radiation group was subdivided into proton and photon therapy cohorts. Univariate and Kaplan–Meier analyses assessed demographic, clinical, and survival outcomes. Multivariate Cox proportional hazards models identified prognostic factors, and survival predictive models were evaluated using Area Under the Curve (AUC) metrics.

**Results:**

Of 1971 patients, 343 (17.4%) received radiation. Surgery was less common in the radiation group (53.9% vs 82.6%, *P* < .001). Combined surgery and radiation had the best survival outcomes, with proton therapy showing superior survival to photons (*P* < .001). High-dose radiation (Biologically Effective Dose [BED] >70 Gy) and Stereotactic Body Radiation Therapy (SBRT) improved survival (*P* < .001). Surgery was associated with increased mortality risk (hazard ratio [HR] = 0.35, *P* < .001), while radiation showed increased risk (HR = 1.31, *P* = .003). Machine learning identified tumor size thresholds of 75 mm for photon and 70 mm for proton therapy as predictive of mortality. DeepSurv (AUC = 0.708) identified distant metastasis, tumor size, and age as important prognostic factors for 10-year survival.

**Conclusion:**

Gross total resection (GTR) is the most effective treatment for spinal chondrosarcoma. High-dose radiation therapy (BED > 70 Gy) can be combined with surgery to improve survival in advanced cases. Proton therapy offers superior long-term survival compared to photons, and dose-escalated techniques (Stereotactic Radiosurgery [SRS] and Intensity-modulated radiation therapy [IMRT]) show potential in enhancing outcomes.

Key Points

- Gross total resection (GTR) remains crucial for managing spinal chondrosarcoma.
- Proton therapy improves long-term survival compared to photon therapy.
- High-dose radiation (Biologically Effective Dose > 70 Gy) enhances survival outcomes in advanced cases.

Importance of the Study

This study addresses critical gaps by providing population-level insights into the management of spinal chondrosarcoma, a rare and aggressive malignancy, and explores the optimal treatment strategy and the role of radiation, which remains limited in the literature. Gross total resection (GTR) remains the primary treatment. Proton provided superior long-term survival benefits compared to photon therapy. High-dose radiation (Biologically Effective Dose >70 Gy) significantly improves patient outcomes, especially when combined with surgery. The study also highlights the potential of dose-escalated techniques like SRS and IMRT to enhance survival, advocating for research in more advanced technologies. Machine learning models predicting mortality based on tumor size and metastasis offer a novel, personalized approach to treatment planning. Future studies should validate optimal radiation doses, assess proton therapy in larger cohorts, and standardize protocols for combining surgery with advanced radiation to improve survival and reduce toxicity.

Spinal chondrosarcomas are rare yet aggressive malignant cartilage tumors of the bone with substantial management challenges.^1^ These tumors often present with progressive localized back pain and/or neurologic deficits. Despite their rarity, these tumors have a high morbidity and poor prognosis.^2^ The incidence of chondrosarcomas is approximately 1 per million population annually, with spinal involvement reported in 6.5%-10% of cases and sacral involvement in about 5%.^3^ Data from the Surveillance, Epidemiology, and End Results (SEER) database report 2-, 5-, and 10-year survival rates of 63%, 53%, and 45%, respectively, with the median survival approximately of 6.9 years, with a disease-specific survival rate of 58% at 10 years.^4^^,^^5^ These outcomes are often compounded by the tumor’s tendency for recurrence and the morbidity associated with its management, necessitating long-term monitoring and follow-up.^6–8^

The current standard of care for spinal chondrosarcomas of the mobile spine and sacrum involves *en bloc* resection with the goal of achieving negative surgical margins.^9^ However, the complex anatomy of the spine and surrounding critical structures often precludes gross total resection (GTR) in many cases. This anatomical limitation highlights the importance of adjuvant therapies, particularly radiation therapy (RT), despite the well-documented radioresistance and chemoresistance of chondrosarcomas.^10^ Photon and proton RT have emerged as valuable adjuvant treatment modalities for spinal chondrosarcoma, particularly in the context of local control and symptom management following surgical resection.^10–12^ High-dose proton therapy, in particular, has shown promise when compared with historical data on photon RT and traditional RT approaches, offering improved outcomes for local control.^13^

Despite these advancements, the small sample sizes in the existing literature and the absence of robust comparative trials have limited the establishment of clear superiority between photon and proton RT. Similarly, key questions regarding the optimal RT dosage, beam delivery technology, and timing (adjuvant versus neoadjuvant) remain unanswered in the current literature. Our study aims to compare long-term survival outcomes between proton and photon RT as treatments using the National Cancer Database (NCDB). Additionally, it seeks to identify the most effective RT strategies for managing patients with spinal chondrosarcomas.

## Methods

### Cohort Selection and Ethics

The NCDB is a comprehensive US cancer registry supporting multi-center research through data from Commission on Cancer-accredited facilities. All spinal malignancy cases were identified based on the primary site of C41. 2 (vertebra) and C41.4 (sacrum and coccyx) per ICD-O-3 codes from the NCDB for the period between January 1, 2004, and December 31, 2017. Among which, patients with histologically confirmed spinal chondrosarcoma were identified based on the ICD-O-3 codes: 9220, 9221, 9231, 9240, 9242, and 9243. This study adhered to HIPAA regulations, followed STROBE guidelines for observational studies, and was deemed exempt from formal IRB approval upon evaluation, as it utilized only de-identified data and did not necessitate informed consent.

### Patient Demographics and Disease Characteristics

Patient demographics and baseline health conditions included age, sex, race, insurance status, comorbidities (evaluated using the Charlson–Deyo Comorbidity Classification scores of 0, 1, or ≥2), tumor size (recorded as the largest diameter in millimeters), and distant metastasis status at diagnosis.

### Treatment Characteristics

Treatments in this study included surgery, RT, and chemotherapy. The study population was categorized into radiation and no radiation groups based on RT status. For surgically treated cases, the extent of resection (EOR) was specified as GTR or subtotal resection (STR). Chemotherapy regimens (single- or multi-agent) were recorded. The radiated population was further categorized into photon or proton groups. Beam technologies and sequential types (adjuvant or neoadjuvant) were reported. Biologically Effective Dose (BED) was calculated by BED = *n* · *d* · (1 + *d*/(α/β)) according to Fowler, where *n* is the number of fractions, *d* the dose per fraction, and α and β the coefficients of the linear quadratic cell model. The α/β value for chondrosarcoma was assumed to be 2.45 Gy.^14^^,^^15^ The study population was further stratified into low dose (BED ≤ 70 Gy) and high dose (BED > 70 Gy) groups.^16^^,^^17^ The dose and modality stratification also provides an indirect distinction between palliative and curative regimens, which is not explicitly documented in NCDB. Additionally, the duration from diagnosis to the initiation of radiation was also documented.

### Primary and Secondary Outcome

The primary outcome of the study was 10-year overall survival (OS), defined as the time from diagnosis to 120 months. Secondary outcomes included mortality at 90 days, 1 year, 5 years, and last follow-up (>15 years); the length of stay (LOS) in surgical admission; readmission within 30-day rate, and palliative care rate.

### Statistical Analysis

Categorical variables were documented as frequencies and percentages and analyzed using chi-square or Fisher’s exact tests. Continuous variables were documented as means with SDs and assessed using independent samples *t*-tests for normal distributions or Mann–Whitney *U*-tests for non-normal distributions. A significance level of *P* < .05 was applied. Univariate analyses were conducted to compare patient baseline and disease characteristics and treatment approaches between groups. Kaplan–Meier (KM) survival analysis, with log-rank tests, was performed to evaluate the primary outcome, 10-year OS. Multivariate Cox proportional regression model was performed in the overall population and the radiated populations and was also repeated in the subpopulation without distant metastasis at diagnosis to address the impact of including metastatic cases. Results were reported in hazard ratio (HR), 95% CIs, and *P*-values.

The data was imputed using the MissForest algorithm, a robust nonparametric method capable of handling both categorical and continuous variables simultaneously. The dataset was partitioned into training (80%) and testing (20%) subsets, with class imbalance addressed through the Synthetic Minority Over-sampling Technique (SMOTE), which generated synthetic samples for underrepresented classes. SMOTE was chosen for its proven efficacy in mixed oncology datasets, generating realistic synthetic samples via interpolation to reduce bias and enhance generalization better than basic over/undersampling.^18^^,^^19^

We employed 5 survival prediction models for patients treated with radiation for spinal chondrosarcomas to assess the optimal cutoffs for tumor size (measured in millimeters) across different radiation modalities (protons and photons) and beam technologies at 120 months that delineated mortality risk, using the best-performing model with the largest area under the receiver operating characteristic curve (AUC). We also employed 7 survival prediction models to predict the risk of 10-year mortality. Hyperparameter tuning was performed via grid search, and time-dependent AUC curves assessed model discrimination over 160 months. The model with the highest AUC at 120 months was selected for further analysis. Feature importance was quantified using absolute coefficient values (ACV), ranking clinical variables by predictive contribution. Model performance was validated through calibration curves and KM survival analyses to ensure robustness and reliability. For Cox models, the proportional hazards assumption was verified using Schoenfeld residuals tests, with all *P*-values >.05 indicating no violations (eg, *P* = .34 for comorbidity, *P* = .21 for distant metastasis). Machine learning model calibration was evaluated via calibration curves, plotting predicted probabilities against observed frequencies in bins; the curve aligned closely with the 45-degree line, confirming good calibration despite limited bin counts in some areas.

All analyses were performed using R Studio (v4.4.2), Python 3.8.12 with scikit-learn (0.24.2) and lifelines (0.26.0).

## Results

### Patient Demographics and Baseline Disease Characteristics

The study cohort included 1971 patients diagnosed with spinal chondrosarcoma from the NCDB database, with 343 (17.4%) receiving RT and 1628 (82.6%) managed without RT (Figure S1, Table 1). The mean age of the entire cohort was 54.1 ± 16.8 years, with patients in the radiation group being significantly older than those in the non-radiation group (59.4 ± 17.6 years vs 53.0 ± 16.4 years, *P < *.001). Sex or racial distribution did not differ significantly between groups, with male and White groups being predominant, respectively. Insurance status differed significantly between groups (*P* < .001): private insurance was more common among patients in the non-radiation group (58.5% vs 44.6%), whereas Medicare coverage was notably higher in the radiation group (41.4% vs 27.4%). The mean tumor size for the entire cohort was 113.9 ± 131.2 mm, with no significant difference between the radiation and non-radiation groups. Patients with a comorbid score of 0 were more prevalent in the non-radiation group compared to the radiation group (85.0% vs 79.0%); in contrast, patients with a comorbid score of 1 or ≥2 were more common in the radiation group (14.3% vs 11.1% and 6.7% vs 3.9%, respectively, *P* = .014). Distant metastasis at diagnosis was significantly more frequent in the radiation group compared to the non-radiation group (22.4% vs 6.3%, *P *< .001), among which the lungs were the most common location in both groups (15% vs 4.7%, *P* < .001), followed by bones (7.9% vs 2.6%, *P* = .007), while brain and liver were comparably rare in both groups.

**Table 1.** Demographics, disease and treatment characteristics and clinical outcomes of patients with spinal chondrosarcoma treated with radiation versus no radiation

	Total (*N* = 1971)	No radiation (*N* = 1628)	Radiation (*N* = 343)	*P-value*
**Age**				
Mean ± SD (years)	54.1 ± 16.8	53.0 ± 16.4	59.4 ± 17.6	<.001
**Sex**				.635
Male	1190 (60.4%)	979 (60.1%)	211 (61.5%)	
Female	781 (39.6%)	649 (39.9%)	132 (38.5%)	
**Race**				.708
White	1542 (79.6%)	1271 (79.5%)	271 (79.9%)	
Black	153 (7.9%)	127 (7.9%)	26 (7.7%)	
Asian	62 (3.2%)	50 (3.1%)	12 (3.5%)	
Hispanic	153 (7.9%)	130 (8.1%)	23 (6.8%)	
Other	27 (1.4%)	20 (1.3%)	7 (2.1%)	
N-Miss	34	30	4	
**Insurance status**				<.001
Private insurance	1055 (56.1%)	905 (58.5%)	150 (44.6%)	
Medicare	562 (29.9%)	423 (27.4%)	139 (41.4%)	
Medicaid	168 (8.9%)	141 (9.1%)	27 (8.0%)	
Other government	29 (1.5%)	20 (1.3%)	9 (2.7%)	
Not insured	68 (3.6%)	57 (3.7%)	11 (3.3%)	
N-Miss	89	82	7	
**Tumor size**				
Mean ± SD (mm)	113.86 ± 131.22	115.44 ± 132.10	104.76 ± 126.02	.285
**Comorbid score**				.014
0	1654 (83.9%)	1383 (85.0%)	271 (79.0%)	
1	230 (11.7%)	181 (11.1%)	49 (14.3%)	
≥2	87 (4.4%)	64 (3.9%)	23 (6.7%)	
**Distant metastasis at diagnosis**				<.001
Yes	143 (9.1%)	83 (6.3%)	60 (22.4%)	
Lung	55 (6.3%)	34 (4.7%)	21 (15.0%)	<.001
Bone	30 (3.4%)	19 (2.6%)	11 (7.9%)	.007
Brain	2 (0.2%)	1 (0.1%)	1 (0.7%)	.365
Liver	7 (0.8%)	4 (0.5%)	3 (2.1%)	.138
No	1439 (91.5%)	1231 (93.7%)	208 (77.6%)	
N-Miss	399	314	75	
**Surgery binary**				<.001
Yes	1529 (77.6%)	1344 (82.6%)	185 (53.9%)	
No	441 (22.4%)	283 (17.4%)	158 (46.1%)	
N-Miss	1	1	0	
**EOR**				<.001
GTR	978 (55.3%)	925 (62.7%)	53 (18.2%)	
STR	351 (19.9%)	271 (18.4%)	80 (27.5%)	
N-Miss	200	148	52	
**Chemotherapy**				<.001
Yes	180 (9.3%)	123 (7.7%)	57 (16.7%)	
Single-agent	24 (1.2%)	12 (0.8%)	12 (3.5%)	
Multi-agent	156 (8.1%)	111 (7.0%)	45 (13.2%)	
No	1757 (90.7%)	1473 (92.3%)	284 (83.3%)	
N-Miss	34	32	2	
**Clinical outcomes**				
**Length of stay**				
Mean ± SD (days)	10.1 (12.3)	10.4 (12.3)	7.8 (12.4)	.015
**Palliative care rate**	67 (3.5%)	25 (1.6%)	42 (12.4%)	<.001
**30-Day readmission rate**	114 (5.8%)	101 (6.2%)	13 (3.8%)	.082
Mortality rates				
90-day	79 (4.4%)	56 (3.8%)	23 (7.3%)	.005
1-year	267 (14.8%)	181 (12.1%)	86 (27.3%)	<.001
5-year	613 (33.9%)	434 (29.1%)	179 (56.8%)	<.001
10-year	697 (38.6%)	507 (34.0%)	190 (60.3%)	<.001
LFU	710 (39.3%)	515 (34.5%)	195 (61.9%)	<.001

Abbreviations: EOR, extent of resection; GTR, gross total resection; LFU, Last Followup; STR, subtotal resection.

The radiated population included 335 patients, with 301 (89.9%) receiving photons and 34 (10.1%) receiving protons (Table 2). No significant difference was observed in the mean age, sex, racial, or insurance coverage distribution. The comorbidity distribution and mean tumor size were also similar between groups. Distant metastasis at diagnosis was more frequently presented in patients treated with photons (24.2% vs 7.4%, *P* = .048).

**Table 2.** Demographics, disease and treatment characteristics and clinical outcomes of patients with spinal chondrosarcoma treated with radiation between the photon and proton group

	Total (*N* = 335)	Photons (*N* = 301)	Protons (*N* = 34)	*P-value*
**Age**				
Mean ± SD (years)	59.6 ± 17.61	60.2 ± 17.4	54.4 ± 18.9	.070
**Sex**				.707
Male	207 (61.8%)	187 (62.1%)	20 (58.8%)	
Female	128 (38.2%)	114 (37.9%)	14 (41.2%)	
**Race**				.424
White	266 (80.4%)	240 (80.3%)	26 (81.2%)	
Black	25 (7.6%)	21 (7.0%)	4 (12.5%)	
Asian	12 (3.6%)	11 (3.7%)	1 (3.1%)	
Hispanic	22 (6.6%)	22 (7.4%)	0 (0.0%)	
Other	6 (1.8%)	5 (1.7%)	1 (3.1%)	
N-Miss	4	2	2	
**Insurance status**				.069
Not insured	11 (3.3%)	11 (3.7%)	0 (0.0%)	
Private insurance	147 (44.5%)	128 (43.2%)	19 (55.9%)	
Medicaid	27 (8.2%)	26 (8.8%)	1 (2.9%)	
Medicare	136 (41.2%)	125 (42.2%)	11 (32.4%)	
Other government	9 (2.7%)	6 (2.0%)	3 (8.8%)	
N-Miss	5	5	0	
**CDCC comorbidity score**				.831
0	264 (78.8%)	238 (79.1%)	26 (76.5%)	
1	48 (14.3%)	42 (14.0%)	6 (17.6%)	
2+	23 (6.9%)	21 (7.0%)	2 (5.9%)	
**Tumor size**				
Mean ± SD (mm)	101.70 ± 116.54	105.17 ± 121.62	68.47 ± 29.76	.192
**Metastasis at diagnosis**				.048
Yes	59 (22.4%)	57 (24.2%)	2 (7.4%)	
No	204 (77.6%)	179 (75.8%)	25 (92.6%)	
N-Miss	72	65	7	
**Radiation regimen**				.543
Radiation standalone	154 (46.1%)	140 (46.7%)	14 (41.2%)	
Radiation with surgery	180 (53.9%)	160 (53.3%)	20 (58.8%)	
N-Miss	1	1	0	
**Radiation sequence**				.024
Adjuvant	153 (84.5%)	136 (84.5%)	17 (85.0%)	
Neoadjuvant	22 (12.2%)	21 (13.0%)	1 (5.0%)	
Neoadjuvant and adjuvant	5 (2.8%)	4 (2.5%)	1 (5.0%)	
Intraoperative	1 (0.6%)	0 (0.0%)	1 (5.0%)	
N-Miss	154	140	14	
**Duration from diagnosis to the initiation of radiation**				<.001
Mean ± SD (days)	72.2 ± 63.0	68.3 ± 58.6	111.2 ± 88.5	
**Beam technology**				.018
Conformal (3-D) therapy	25 (7.5%)	25 (8.4%)	0 (0.0%)	
IMRT	290 (87.1%)	256 (85.6%)	34 (100.0%)	
SBRT	17 (5.1%)	17 (5.7%)	0 (0.0%)	
N-Miss	3	3	0	
**Biologically effective dose**				
Mean ± SD (Gy)	89.5 ± 36.2	88.9 ± 36.3	99.0 ± 33.6	.294
Biologically effective dose strata				.230
≤ 70 Gy	66 (26.6%)	64 (27.5%)	2 (13.3%)	
>70 Gy	182 (73.4%)	169 (72.5%)	13 (86.7%)	
N-Miss	87	68	19	
Clinical outcomes				
**Length of stay**				
Mean ± SD (days)	7.8 ± 12.5	8.3 ± 13.1	3.9 ± 4.1	.207
**30-Day readmission rate**	13 (3.9%)	11 (3.7%)	2 (5.9%)	.524
**Palliative care rate**	42 (12.6%)	42 (14.0%)	0 (0.0%)	.020
**Mortality rate**				
90-day	23 (7.4%)	23 (8.3%)	0 (0.0%)	.090
1-year	85 (27.5%)	85 (30.7%)	0 (0.0%)	<.001
5-year	177 (57.3%)	169 (61.0%)	8 (25.0%)	<.001
10-year	188 (60.8%)	177 (63.9%)	11 (34.4%)	.001
LFU	192 (62.1%)	180 (65.0%)	12 (37.5%)	.002

Abbreviation: CDCC, Charlson–Deyo Comorbidity Classification; IMRT, Intensity-Modulated Radiation Therapy; LFU, Last Follow-up; SBRT, Stereotactic Body Radiation Therapy.

### Treatment Characteristics

Significant differences in treatment characteristics were observed between the radiation and no radiation groups (Table 1). Surgical treatment was significantly less common in the radiation group compared to the non-radiation group (53.9% vs 82.6%, *P* < .001). Among surgically treated cases, GTR was achieved in a larger proportion of patients without radiation (62.7% vs 18.2%, *P* < .001), while STR was more common in the radiation group (27.5% vs 18.4%, *P* < .001). Chemotherapy was significantly more common in the radiation group compared to the non-radiation group (16.7% vs 7.7%, *P* < .001), with multi-agent being the most prevalent regimen (13.2% vs 7.0%, *P* < .001) compared to a single-agent regimen.

Within the radiated population, both photons and protons were more prevalently used in combination with surgery rather than as a standalone treatment, predominantly as an adjuvant therapy instead of a neoadjuvant or intraoperative therapy (Table 2). Intensity-modulated radiation therapy (IMRT) was adopted in all proton cases (intensity-modulated proton therapy [IMPT]) (100%) and in the majority of photon cases (85.6%, *P* = .018), while conformal (3-D) therapy and Stereotactic Body Radiation Therapy (SBRT) were also used for photons. The average BED was 89.5 ± 36.2 Gy for the overall cohort, with 73.4% of patients receiving BED > 70 Gy; no difference was detected between photon and proton groups. Additionally, patients treated with protons experienced a significantly longer duration from diagnosis to the initiation of radiation compared to photon patients (111.2 ± 88.5 days vs 68.3 ± 58.6 days, *P* < .001).

### Clinical Outcomes

The radiation and no radiation groups showed significantly different clinical outcomes (Table 1). Patients treated with radiation experienced significantly shorter mean LOS compared to the non-radiation group (7.8 ± 12.4 days vs 10.4 ± 12.3 days, *P* = .015). The rate of palliative care utilization was significantly higher in the radiation group (12.4%) compared to the non-radiation group (1.6%, *P* < .001), whereas the 30-day readmission did not show a statistical difference. In terms of survival, the radiation group showed significantly higher short-term mortality rate at 90 days (7.3% vs 3.8%, *P* = .005) and long-term intervals at 1 year (27.3% vs 12.1%, *P* < .001), 5 years (27.3% vs 12.1%, *P *< .001), 10 years (60.3% vs 34.0%, *P *< .001), and last follow-ups (61.9% vs 34.5%, *P* < .001). Within the radiated population, short-term mortality did not differ significantly; nevertheless, the proton group showed significantly lower mortality at long-term intervals at 1 year (0% vs 30.7%, *P* < .001), 5 years (25% vs 61%, *P < *.001), 10 years (34.4% vs 63.9%, *P* = .001), and last follow-ups (37.5% vs 65%, *P = *.002).

**Figure 1.**
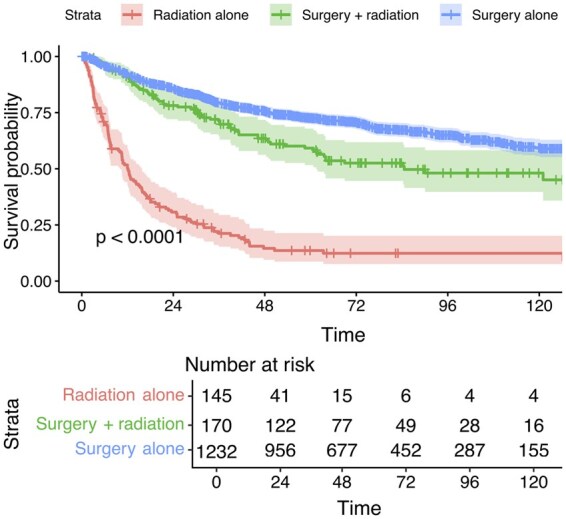
The impact of treatment approaches on the 10-year OS of patients with spinal chondrosarcoma.

**Figure 2.**
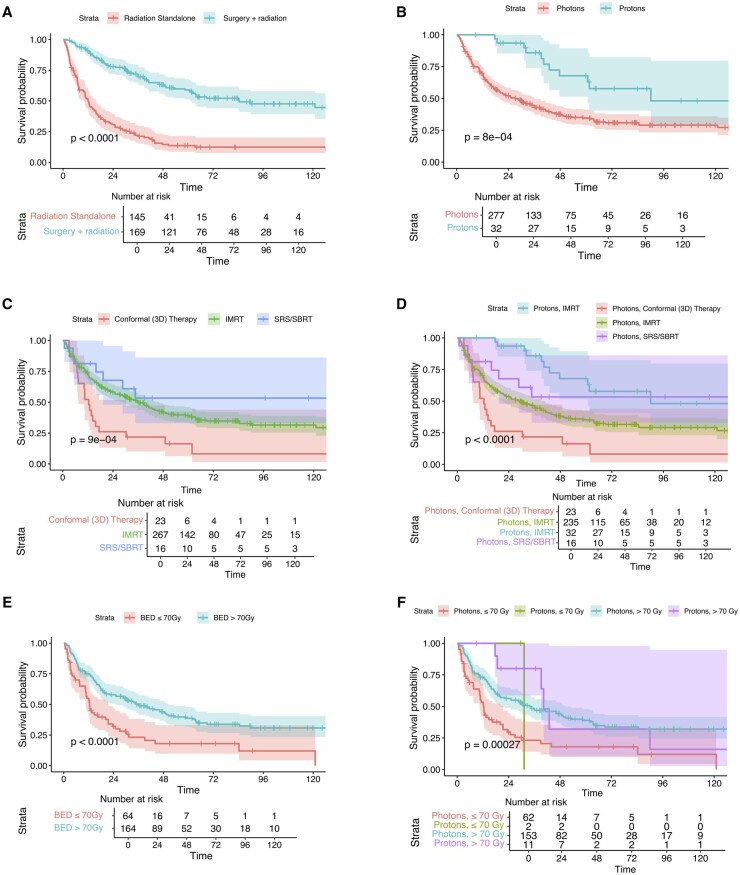
The impact on 10-year OS of patients with spinal chondrosarcoma of (A) radiation-surgery modality; (B) photon versus proton; (C) beam technologies; (D) proton/photon-stratified beam technologies; (E) BED strata; and (F) proton/photon-stratified BED strata.

Kaplan–Meier survival analysis demonstrated significant differences in 10-year OS across treatment modalities. Overall, surgery alone yielded the best OS, followed by surgery with radiation, while radiation alone was associated with the poorest OS (Figure 1; *P* < .0001). Among radiated patients, radiation used in combination with surgery had significantly improved OS compared to radiation as a standalone treatment (Figure 2A; *P* < .0001). Proton therapy demonstrated remarkably superior OS compared to photons (Figure 2B; *P* = .0008). In terms of beam technologies, SBRT was the best approach overall, followed by IMRT and conformal therapy (Figure 2C; *P* = .0009). When stratified by photon vs proton, IMPT was associated with the best OS, while among photon cases, SBRT was superior to IMRT and conformal therapy (Figure 2D; *P < *.0001). Radiation dose significantly impacted OS: high-dose radiation (BED >70 Gy) showed better survival compared to the low-dose group (Figure 2E; *P < *.0001). Stratified analysis revealed that both high-dose and low-dose protons demonstrated better short-term mortality compared to their photon counterparts, but the advantages were diminished after 24 months due to low numbers of proton cases (Figure 2F; *P* = .0004).

### Prediction for Mortality Risk and Identifications of Clinical Thresholds

**Figure 3.**
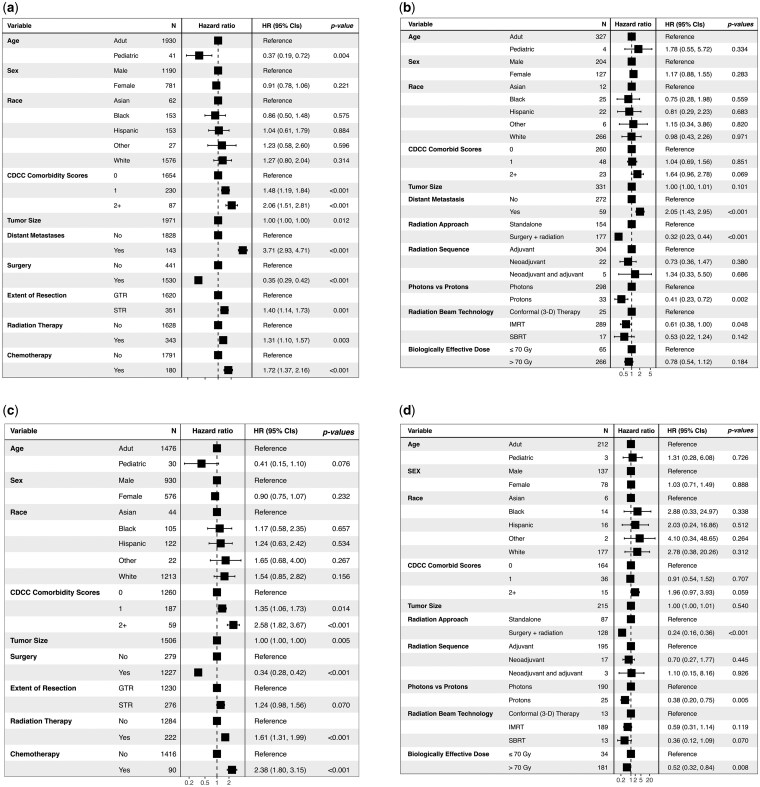
Cox proportional hazards models of (A) the overall population with spinal chondrosarcoma and (B) the radiated population with spinal chondrosarcoma; (C) the population without distant metastases; and (D) the population without distant metastases treated with radiation.

The multivariate Cox regression models were performed in the entire and radiated populations. Within the overall population, higher comorbidity scores (score 1: HR = 1.48, *P < *.001; scores 2+: HR = 2.06, *P < *.001) and presence of distant metastasis at diagnosis (HR = 3.71, *P < *.001) were associated with higher mortality risk. Patients treated with surgery were associated with significantly lower mortality compared to their no-surgery counterparts (HR = 0.35, *P < *.001); in contrast, patients treated with radiation and chemotherapy were at high mortality risk (HR = 1.31, *P = *.003; HR = 1.72, *P < *.001, respectively) (Figure 3A). Similar results were also observed within the subpopulation without distant metastasis (Figure 3C). Within the radiated population, distant metastasis (HR = 2.05, *P < *.001) remained a risk factor for mortality (Figure 3B). Radiation in combination with surgery was associated with remarkably lower risk compared to radiation alone (HR = 0.32, *P < *.001). Protons were associated with significantly lower risk than photons (HR = 0.41, *P = *.002), and IMRT was associated with lower risk compared to other beam technologies (HR = 0.61, *P = *.048). Within the radiated subpopulation without distant metastasis, combined surgery and radiation (HR: 0.24, *P < *.001) and protons (HR: 0.38, *P < *.005) remained protective, high-dose radiation emerged as a protective factor associated with lower mortality risk (HR: 0.52, *P < *.008) (Figure 3D). Notably, in both the overall population and the non-metastatic subpopulation, STR with radiation was associated with inferior survival compared to GTR, indicating that adjuvant radiation did not offset the survival disadvantage of STR. Sex, race, or tumor size did not show significant association with long-term mortality in either population.

### Machine Learning and Deep Learning for Mortality Risk Prediction

**Figure 4.**
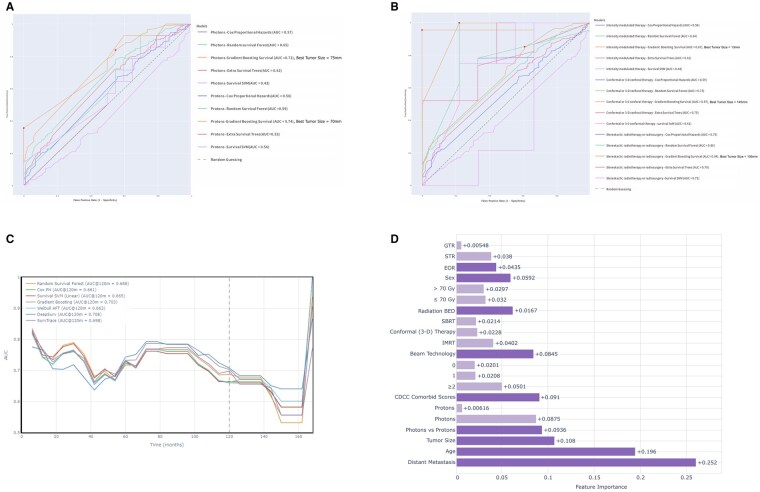
Machine learning-based prediction of long-term mortality risk using tumor size as a predictor across (A) photons versus protons and (B) beam technologies; (C) ROC analysis of machine learning models in predicting long-term mortality; and (D) deepSurv-based SHAP features for risk factor assessment.

Multiple machine-learning-based models to predict 10-year mortality utilizing tumor size as a predictor were evaluated and compared between radiation modality (protons vs photons) and among beam technologies separately. The ROC analysis identified the Gradient Boosting Classifier (GBC) model to be the best-performing model with the largest AUC, based on which a tumor size threshold of 75 mm for photon-treated cases (GBC: AUC = 0.72) and 70 mm for proton-treated cases (GBC: AUC = 0.74) was identified that delineated distinct mortality risk (Figure 4A). For beam technologies, tumor size cutoffs were identified as: 13 mm for IMRT (GBC: AUC = 0.67), 145 mm for conformal therapy (GBC: AUC = 0.97), and 105 mm for SBRT (GBC: AUC = 0.94) (Figure 4B).

We implemented a comprehensive machine learning analysis to identify risk factors associated with 10-year mortality risk within the radiated population. Among the compared survival models at 120 months of follow-up, the DeepSurv achieved the highest discriminative performance (AUC = 0.708), followed by Gradient Boosting (AUC = 0.703) (Figure 4C). SHAP feature importance analysis based on DeepSurv revealed that the presence of distant metastasis (ACV = 0.252) was the strongest predictor of mortality risk, followed by age (ACV = 0.196) and tumor size (ACV = 0.108). Photons were more predictive of high long-term mortality compared to protons (ACV = 0.0875) (Figure 4D). High comorbidity scores (2+) were also more predictive of high mortality risk compared to low or no comorbidities. Radiation beam technology and radiation dose demonstrated less predictive value, while sex and EOR showed minimal impact on mortality prediction. Proportionality checks for Cox models showed no significant violations (all Schoenfeld residuals *P* > .05). Calibration curves for machine learning models demonstrated reliable predictions, with observed means tracking predicted probabilities (eg, at predicted ∼0.46, observed = 0.33; at ∼0.69, observed = 1.00).

## Discussion

Spinal chondrosarcomas are rare spinal tumors that require complex management due to local invasiveness and limited systemic treatment options. Our study showed that surgery, particularly GTR, remains crucial for improving survival. Radiation was overall associated with poorer outcomes, presumably attributed to the lower GTR rate and higher frequency of distant metastasis observed within the irradiated population. Disparities in clinical and socioeconomic characteristics between radiated and no-radiation populations were observed. Within the radiated population, protons were superior to photons in conferring survival benefits, and Stereotactic Radiosurgery (SRS) was associated with the best OS compared to other beam technologies. The sequence of radiation did not lead to varied survival. Machine learning models demonstrated that distant metastasis, age, and tumor size are top prognostic predictors of high long-term mortality. The study reinforces the importance of surgery in managing spinal chondrosarcoma and highlights the need for high-dose RT to optimize care.

Current guidelines established surgery as the primary management for spinal tumors and that aggressive resection achieving negative margins is critical for clinical outcomes, which is supported by our study.^20^ The poorer survival of the radiation population observed in our study can be associated with multiple factors. First, the high prevalence of distant metastasis at diagnosis observed in the radiation group reflects a more advanced disease in this group, thus limiting the applicability of complete surgical resection, as also observed in our study, particularly the low rate of GTR. Also, the radioresistance of chondrosarcoma due to its distinct composition of dense cartilaginous extracellular matrix generally requires a higher dose to be efficacious, which is usually restrained by the spinal cord tolerance and has been found to be associated with significant late toxicity and long-term sequelae.^17^^,^^21^ In addition, socioeconomic disparities were observed in our study, wherein patients in the radiation group were more likely to be covered by Medicare, which has been found to be associated with lower odds of receiving surgery by a large-scale nationwide study by Tang et al. in 2024.^22^^,^^23^ Furthermore, even when adjusting for metastatic status, STR combined with radiation did not confer survival equivalent to GTR. This finding underscores that complete resection remains the critical determinant of long-term survival, while STR plus adjuvant radiation cannot compensate for the survival disadvantage of incomplete resection. These findings reinforce the necessity of immediate and complete surgical interventions, whenever deemed to be feasible, to optimize survival outcomes in spinal chondrosarcoma patients.

In managing advanced disease when radiation is necessary, radiation alone was found insufficient in conferring survival benefits compared to radiation used as an adjuvant to surgery, which was consistent with findings summarized in a review by Bian et al. in 2016.^21^ Similar results were also found in previous studies, as Kuo et al.^20^ in 2024 proposed the role of radiation in achieving disease control in non-resectable cases and Catanzano et al.^24^ in 2019 showed radiation to be only beneficial in surgically-treated cases with positive margins. Our study also demonstrated the superiority of protons over photons in treating spinal chondrosarcoma, which remained scarce in current literature. Proton therapy has been increasingly favored in managing certain spinal tumors, reported to be associated with fewer long-term complications in multiple studies.^25^^,^^26^ A prospective clinical trial found high-dose proton therapy (up to 79 Gy) to be safe and effective in treating spinal and skull base chondrosarcoma due to its capacity to deliver escalated and conformal doses that better spare critical structures.^27^ This advantage of dose-escalation of protons was also reflected in our study, consistent with several studies.^10^^,^^13^^,^^28^^,^^29^

Notably, access to proton beam therapy remains limited due to disparities in age, race, socioeconomic status, and insurance coverage.^30^ Burus et al.^31^ in 2024 reported that the drive-time to proton facilities varied substantially among the US population, with older patients and patients living in rural areas with poverty facing extended travel time. These findings highlight the need to address socioeconomic disparities to mitigate gaps in healthcare access and to make proton therapy more accessible.

In terms of the beam technologies, SBRT/SRS was found to be the most superior beam technology in treating spinal chondrosarcoma in our study. SRS is an emerging technique favored for managing spine tumors due to its advantages in delivering conformal doeses to the targeted volume with minimal toxicity.^20^^,^^32^ It has been conventionally recommended for palliating metastatic spine tumors, but its role in managing primary tumors has been increasingly highlighted.^33^ Multiple studies reported SRS to be effective in treating chondrosarcoma, and Sherry et al. in 2023 identified a single-fraction SRS (24 Gy) as safe and effective in managing spinal chondrosarcoma.^34–36^ Moreover, all the SBRT/SRS cases in our study were photon-based; however, a review by Amichetti et al.^37^ in 2012 proposed the potential of proton-SRS in skull base tumors and gaps in the context of chondrosarcoma that are in need for further research. IMRT also demonstrated survival benefits compared to conventional 3-D conformal therapy, which is also in line with current literatures.^17^^,^^24^^,^^38–40^ Among which, IMPT showed promising results in our study, which is also supported by a recent study by Miladinovic et al. in 2024, wherein IMPT was found effective in managing skull base chondrosarcoma with acceptable toxicities.^17^^,^^41^

Radiation dosage is shown to be a critical parameter that impacts OS in our study. According to current literature, high-dose radiation is generally required to manage chondrosarcomas due to its astonishing radio resistance. Multiple studies have established a consensus of 70 Gy as the minimum threshold for therapeutic efficacy.^27^^,^^42^ Palm et al.^43^ in 2019 specifically investigated this threshold and showed that patients who received radiation doses exceeding 70 Gy had a higher 5-year survival rate compared to those who received lower doses (<70 Gy). Our studies supported this finding and further demonstrate that high-dose radiation provides sustained benefits, being associated with a reduced 10-year mortality risk.

On the other hand, the sequential type of radiation (adjuvant, neoadjuvant, or intraoperative) did not have a significant impact on survival in our study, which is consistent with previous studies suggesting that the sequence of radiation relative to surgery may not be as critical as achieving optimal resection margins and delivering an adequate radiation dose.^44^ A few studies reported the use of intraoperative radiation therapy (IORT) in managing spinal metastases; given that spinal chondrosarcoma without *en bloc* resection achieved is at particularly high risk of recurrence, IORT may hold value for clearing microscopic residual tumors to prevent tumor recurrence, which is worth to be explored.^45–47^

In the machine learning-based mortality risk prediction of patients with spinal chondrosarcoma, tumor size was employed as a predictor, which has been recognized as a critical prognostic factor for spine tumors in previous literature.^48^ A study by Wang et al.^49^ in 2019 reported a tumor size greater than 10 cm as a predictor for a poor prognosis in patients with bone chondrosarcoma with metastatic disease, while another study by Brown et al.^50^ in 2022 indicated that patients with a tumor size greater than 8 cm were associated with a higher risk of mortality. However, there still lacks a consensus on tumor size threshold that delineates mortality risk, particularly in subpopulations treated with different radiation modalities. Our study identified similar tumor size thresholds for photons (7.5 cm) and protons (7 cm). Although 1 study suggested the potential of protons in treating larger tumors, evidence remains scarce and warrants further investigations.^43^ Tumor size thresholds for different beam technologies were also identified. According to the results, for patients treated with IMRT, patients exceeding a small tumor size of 13 mm were at a higher risk of mortality. In contrast, tumor size thresholds for conformal therapy and SRS were identified to be 145 mm and 105 mm, suggesting their potential in treating large tumors. Overall, tumor size can serve as a valuable tool for clinicians in predicting prognosis for patients with spinal chondrosarcoma and stratifying their mortality risk.

Lastly, the SHAP features, based on the best-performing model, DeepSurv, identified distant metastases as the most important risk factor. Age and tumor size are also critical parameters to predict mortality risk. Photons were more predictive to mortality than protons, which supports the findings in our study. Other parameters, including comorbidities, beam technologies, and radiation dose, are also important factors with predictive values of mortality. These findings highlight the importance of timely diagnosis and intervention before tumor progression, and the need for individualized consideration to improve the clinical outcomes of patients with spinal chondrosarcoma.

### Limitations

The study on spinal chondrosarcomas provides valuable insights but has notable limitations. The reliance on retrospective data from the NCDB introduces potential selection bias and missing variables, including detailed tumor-specific factors such as histologic grade, precise location, and molecular characteristics. While not specifically investigated in this study, future analyses will incorporate granular socioeconomic data to evaluate their impact on treatment and outcomes, particularly in the context of resource-intensive modalities such as proton therapy. A significant limitation is the small sample size for proton therapy, which reduces the statistical power to generalize findings. Variations in radiation protocols across institutions, including fractionation schedules and dosimetric parameters, are not fully accounted for, and the number of fractions delivered per day, an important variable for assessing fractionation schedules, is notably absent. Coding variability is also inherent for large national datasets. Notably, we applied a universal α/β ratio to calculate the BED due to the lack of documentation on the tissues being irradiated, which may diminish the accuracy of the dose and therefore bias the results. External validation is needed to confirm the predictive performance of the machine learning model and the thresholds identified for tumor size and radiation dose, as our use of SMOTE risks overfitting and introducing noise in large registries, potentially inflating training performance, and comparison with alternative approaches such as weighted loss functions, undersampling, or Balanced Random Forest is warranted to preserve data integrity. Calibration analyses were limited by sample size in some bins, potentially affecting precision in subgroups; larger cohorts could refine these assessments. Collaborative efforts with multi-institutional collaborations are currently underway to support independent verification of these findings. Furthermore, the study does not address late toxicities, recurrence patterns, or quality of life outcomes, which are not captured in the national database. As part of an ongoing multi-institutional collaboration, we plan to address this gap by collecting recurrence data and functional outcomes, including pain, motor/sensory deficits, ambulatory status, and bladder/bowel function, to enable a more comprehensive evaluation of treatment impact.

## Conclusion

Surgical resection, particularly GTR, is the most effective treatment for spinal chondrosarcoma. In advanced cases, RT, especially with high doses (BED > 70 Gy), can be combined with surgery to improve survival. Proton therapy offers superior long-term survival compared to photons, and advanced dose-escalated techniques (SRS and IMRT) show potential in enhancing outcomes. Our study highlights the importance of specialized radiation planning, particularly in advanced cases, to optimize treatment outcomes.

## Supplementary Material

vdaf240_Supplementary_Data

## Data Availability

The data used in this study will not be shared or disclosed. All data analyses will be conducted at the author’s institution, and access to the data will be reserved for internal use only.
