# Supplementary figures and images for: Impact of proton vs. photon radiotherapy on overall survival in the management of spinal chondrosarcoma and mortality risk prediction: A nationwide analysis
Source: Neurooncol Adv. 2025 Dec 24;8(1):vdaf240. doi: 10.1093/noajnl/vdaf240 (PMC12812003; doi:10.1093/noajnl/vdaf240)

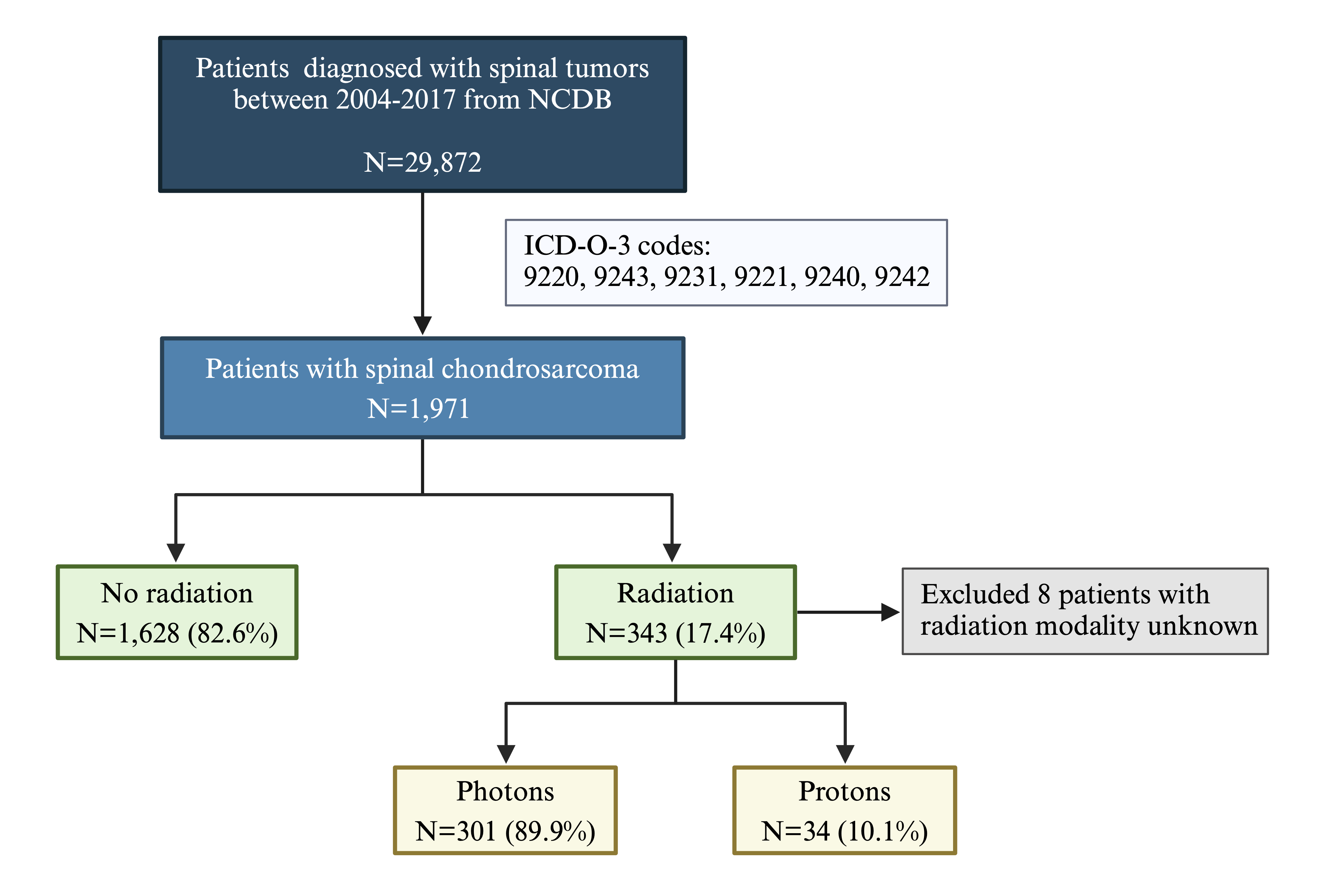

Supplement: vdaf240_Supplementary_Data [file vdaf240_supplementary_data.zip › Supplementary Figure 1.tiff]
